# Antimicrobial Consumption in Medicated Feeds in Vietnamese Pig and Poultry Production

**DOI:** 10.1007/s10393-016-1130-z

**Published:** 2016-05-19

**Authors:** Nguyen Van Cuong, Nguyen Thi Nhung, Nguyen Huu Nghia, Nguyen Thi Mai Hoa, Nguyen Vinh Trung, Guy Thwaites, Juan Carrique-Mas

**Affiliations:** 1 Oxford University Clinical Research Unit, Wellcome Trust Major Overseas Programme, 764 Vo Van Kiet, Ho Chi Minh City, Vietnam; 2 Nuffield Department of Medicine, Oxford University, Oxford, UK; 3 Department of Medical Microbiology, Academic Medical Center, University of Amsterdam, Pietersbergweg 17, 1105 BM Amsterdam, The Netherlands

**Keywords:** antimicrobial, animal production, antimicrobial drug resistance, Vietnam

## Abstract

Antimicrobials are extensively used as growth promoters in animal feeds worldwide, but reliable estimates are lacking. We conducted an internet-based survey of commercial chicken and pig feed products officially approved for sale in Vietnam over the period March–June 2015. Information on the antimicrobial contents in feed products, alongside animal production data, was used to estimate in-feed antimicrobial consumption to produce one kilogram of live animal (chicken, pig), as well as to estimate country-wide antimicrobial consumption through animal feeds. A total of 1462 commercial feed formulations were examined. The survey-adjusted estimated antimicrobial contents were 25.7 and 62.3 mg/kg in chicken and pig feeds, respectively. Overall, it was estimated that 77.4 mg [95% CI 48.1–106.8] and 286.6 mg [95% CI 191.6–418.3] of in-feed antimicrobials were used to raise 1 kg of live chicken and pig, respectively. Bacitracin (15.5% feeds), chlortetracycline (11.4%), and enramycin (10.8%) were the most common antimicrobials present in chicken feed formulations, whereas bacitracin (24.8%), chlortetracycline (23.9%), and florfenicol (17.4%) were the most common in pig feed formulations. Overall, 57% of the total quantitative usage consisted of antimicrobials regarded by WHO of importance for human medicine, including amoxicillin, colistin, tetracyclines, neomycin, lincomycin, and bacitracin. These figures confirm a very high magnitude of in-feed consumption of antimicrobials, especially in pig production. Results from this study should encourage further monitoring of antimicrobials used in animal production, and foster discussion about existing policies on inclusion of antimicrobials in animal feed rations.

## Introduction

Antimicrobial resistance (AMR) is today one of the most important challenges posed to the health community globally (WHO 2014). Antimicrobials are extensively used in veterinary medicine to treat and prevent animal diseases. In addition, in many countries they are often added to feedstuffs as antimicrobial growth promoters (AGP) in order to increase productivity (Page and Gautier 2012). Over the years, there has been mounting evidence that the use of antimicrobials in agriculture (including AGP) contributes to the development of AMR (Alexander et al. 2008; Marshall and Levy 2011; Landers et al. 2012). Although the magnitude of this contribution is unknown, it is likely to be variable depending on the types and quantities of antimicrobials used in each country.

Previous studies have suggested that in Europe and the United States antimicrobials in animal production represent by far the greatest fraction (66–80%) of the total global antimicrobial usage (McEwen and Fedorka-Cray 2002; Moulin et al. 2008). The total amount of antimicrobials used globally for animal production has been estimated to be 63 thousand tons per year. It is expected that it will continue to increase over the next few years due to intensification of livestock production in emerging economies (Van Boeckel et al. 2015). Over recent decades, Vietnam and other countries in the Asia–Pacific region have been experiencing rapid intensification in their livestock production systems; by 2011, this region represented 48% of the global veterinary antimicrobial market (Otte et al. 2012).

There are very few quantitative estimates of the use of AGP in animal feeds. Australian import statistics for 1992–1996 indicated that 55.8% antibiotics imported were for animal feeds, 7.8% for ‘veterinary use,’ and 36.4% for ‘human use’ (Barton 2000). To our knowledge, no formal quantitative–qualitative studies on antimicrobials in animal feed rations in Southeast Asia have been published to date. In Vietnam, the Ministry of Agriculture and Development regularly publishes a list of authorized commercial feed products and trading companies (MARD 2012), as well as an official list of antimicrobials banned for livestock and aquaculture (MARD 2014). We carried out an internet-based survey aimed at investigating the types and quantities of antimicrobials present in commercial feed rations aimed at pig and poultry production in Vietnam. Results were used to extrapolate the total amounts of antimicrobials consumed in pig and chicken production based on official production figures.

## Materials and Methods

### Types and Amounts of Antimicrobials in Commercial Feeds

The target population was ‘commercial feed products aimed at pig and chicken production available in Vietnam during the months March to June 2015.’ To achieve this, all listed feed products destined for poultry and livestock listed in Regulation 26/2012/TT-BNNPTNT (MARD 2012) were individually searched using the Google internet search engine over the period March–June 2015. Through this search, the company responsible for marketing each of the products was identified, as well as its location (province). From each feed formulation, the following information was gathered: antimicrobial type (defined as product with known antibacterial activity), antimicrobial concentration (expressed in mg/kg feed product), and the target species. We excluded ionophore products, mostly aimed at controlling coccidial infections, since they do not seem to encourage antimicrobial resistance (Callaway et al. 2013). The amount of antimicrobial (mean and standard deviation) in feed formulations aimed for pigs and chickens was calculated assuming equal sales of all products. In addition, each feed product was assigned a sampling weight (Dohoo et al. 2003) consisting of the number of provinces from which each company operated. This ensured that feed products distributed by larger companies (i.e., with greater presence in the country) had a higher contribution to the overall survey estimate, since sales of individual products were not known.

### Antimicrobials Used to Produce Chicken and Pig Meat

The amount of each antimicrobial included in feed to produce one typical chicken and one typical pig was estimated by bootstrap simulation (Selvin 1998) based on 100,000 replicates of the product of the following: (1) probability of a feed product containing a given antimicrobial; (2) amounts of antimicrobial present in each feed product; and (3) estimated amount of feed consumed by each animal produced based on the typical weight of Vietnamese chicken and feed breeds and published ‘feed conversion factor’ (FCF) data (Table 1). For feed products with ambiguous labeling (i.e., indicating inclusion of one of two or more listed antimicrobials), each of the antimicrobials was assigned with a random probability inversely dependent on the number of antimicrobials listed. For example, for a feed product indicating the inclusion of either antimicrobial A or B, inclusion of each antimicrobial (A or B) was simulated with a probability of 0.5. These final estimates were converted into the amounts of antimicrobials used to produce one kilogram of pig and chicken meat. To estimate the amount used per week (i.e., intensity of usage), results were divided by the length of the production cycle, since pig and chicken production cycles have different durations.

**Table 1:** Sources of Data Used to Carry Out Bootstrap Simulations to Estimate Amounts of Antimicrobial Consumption Through feed Among Chickens and Pigs in Vietnam.

	Chickens	Pigs	Source
Weight at slaughter (kg) (mean ± SD)	1.77 kg (±0.32)	92.50 kg (±20.0)	Kinh and Hai (2007), FAO (2008)
FCF (mean ± SD)	2.85 (±0.36)	3.90 (±0.78)	Kinh and Hai (2007), FAO (2008)
Duration of production (weeks)	13.0	37.0	Author’s estimates
Annual animal weight production (thousand tons)	545.0^a^	3217.9	GSO (2015)

^a^Assuming that chickens represent 73% of overall poultry production, according to Desvaux and Ton (2008); *FCF* feed conversion factor; *SD* standard deviation.

### Overall Antimicrobial Consumption in Feeds for Chicken and Pig Production

Overall antimicrobial consumption in feeds in Vietnamese pig and chicken production was simply obtained by multiplying the estimated feed consumption (from official annual production figures in kilograms of live animal and FCF) by the amounts (per weight of feed) of antimicrobials based on the feed survey results. The data sources used to perform all analyses are shown in Table 1. All analyses were carried out using R (www.r-project.org). Survey-adjusted estimates were calculated using the ‘survey’ package (Lumley 2004).

## Results

### Internet-Based Survey of Feed Products

A total of 1821 commercial feed products were searched in Google: 1500 listed in Regulation 26/2012/TT-BNNPTNT, plus 321 products identified in the searched companies’ website. Products were marketed by a total of 297 companies located in 39 provinces across Vietnam. A total of 1462 (80.3%) were products aimed at pigs (*n* = 977) and chickens (*n* = 485). Other products were aimed at ducks/Muscovy ducks (*n* = 249), quails (*n* = 60), and cattle/ruminants (*n* = 50). Detailed information about the composition (including data about inclusion of antimicrobials) was available for 1174 (64.4%) feed products. Of those, 513 (43.7%) contained at least one antimicrobial. This percent ranged from 55.4% (377/680) (pig), followed by 42.2% (116/275) (chicken), 18.9% (7/37) (quail), and 9.2% (13/142) (duck/Muscovy duck) feeds. None of the 40 cattle/ruminant feeds with data contained non-ionophore antimicrobials. A total of 5.4% chicken and 21.5% pig feed formulations reported the inclusion of two or more antimicrobials. A number of feed products (10 chicken and 24 for pigs) reported the potential inclusion of one of several antimicrobials (Table 2). Detailed data about the types and quantities of antimicrobials contained in chicken and pig feed products are presented in Table 3.

**Table 2:** Number of Antimicrobials Reported in 1821 Livestock and Poultry Commercial Feeds Investigated.

	Pigs	Chickens	Ducks/muscovy ducks	Quails	Cattle/ruminants	Total
No antimicrobials	303	159	129	30	40	661
1 antimicrobial	231	101	13	7	0	352
1 listed	207	91	13	7	–	318
2 listed	18	10	–	–	–	28
3–5 listed	6	–	–	–	–	6
2 antimicrobials	75	15	0	0	0	90
3 antimicrobials	71	0	0	0	0	71
With antimicrobial	377	116	13	7	0	513
Number of products with data	680	275	142	37	40	1174
All products investigated	977	485	249	60	50	1821

**Table 3:** Description of the Antimicrobial Contents of Chicken and Pig Commercial Feeds Available in the Vietnamese Market.

Antimicrobial	Antimicrobial class	Chickens			Pigs		
		Percent feeds containing (%)		mg/kg (±SD)	Percent feeds containing (%)		mg/kg (±SD)
		Unadjusted	Adjusted [95% CI]		Unadjusted	Adjusted [95% CI]	
Chlortetracycline^b^	Tetracycline	10.9	11.4 [7.4–15.5]	81.1 (±24.9)	29.8	23.9 [20.7–27.2]	62.2 (±15.2)
Bacitracin^c^	Polypeptide	18.5	15.5 [10.9–20.1]	71.1 (±22.3)	13.2	24.7 [16.3–47.9]	42.2 (±12.8)
Colistin^a^	Polymyxin	6.9	7.4 [4.0–10.8]	71.6 (±15.2)	9.3	12.1 [8.9–15.2]	122.4 (±35.8)
Enramycin	Polypeptide	8.0	10.8 [6.1–15.4]	13.2 (±3.8)	5.3	8.0 [5.4–10.7]	35.2 (±8.5)
Tylosin	Macrolide	–	–	–	15.4	10.6 [8.4–12.9]	40.8 (±7.0)
Florfenicol	Amphenicol	–	–	–	7.9	17.4 [13.3–21.4]	96.9 (±22.3)
Lincomycin^c^	Lincosamides	1.1	1.33 [0.5–2.2]	4.0 (±0.0)	0.4	1.1 [0.2–2.0]	54.3 (±14.8)
Oxytetracycline^b^	Tetracycline	–	–	–	1.5	0.9 [0.3–1.5]	54.5 (±7.9)
Neomycin^a^	Aminoglycoside	–	–	–	0.7	0.9 [0.3–1.5]	100.0 (±0.0)
Tiamulin	Pleuromutilin	–	–	–	0.3	0.2 [0–0.4]	119.0 (±3.8)
Avilamycin	Orthosomycine	0.4	0.2 [0.01–0.6]	10.0 (±0.0)	0.7	0.3 [0.1–0.5]	10.0 (±0.0)
Amoxicillin^a^	*β*-lactam	–	–	–	0.7	1.1 [0.2–2.0]	212.0 (±42.5)
Halquinol	Chlorhydroxyquinolone	–	–	–	0.01	0.1 [0–0.1]	180.0 (±0.0)
Tylvalosin	Macrolide	–	–	–	0.1	0.3 [0–0.9]	100.0 (±0.0)
Any antimicrobial		42.2	48.6 [41.7–55. 6]		55.4	58.8 [54.4–63.2]	

The quantitative data only refer to those products reporting the inclusion of each specific antimicrobial.

*CI* confidence interval.

^a^Antimicrobial ‘of critical importance.’

^b^Antimicrobial ‘of high importance.’

^c^Antimicrobial ‘of importance’ (according to WHO 2011).

Antimicrobials considered of ‘critical importance’ for human medicine according to WHO included *β*-lactams (amoxicillin), aminoglycosides (neomycin), and polymyxins (colistin). Antimicrobials considered of ‘high importance’ included tetracyclines (chlortetracycline, oxytetracycline) and lincosamides (lincomycin). Antimicrobials considered ‘of importance’ included polypeptides (bacitracin) (WHO 2011). Tylosin and tylvalosin, both macrolide antimicrobials, were only present in pig feed formulations. After adjusting for company supplier, the proportion of feed products with antimicrobials was 58.8% [95% CI 54.4–63.2] and 48.6% [95% CI 41.7–55.5] for pig and chicken feed products, respectively. Bacitracin (15.5%), chlortetracycline (11.4%), and enramycin (10.8%) were the most common antimicrobials present in chicken feed formulations, whereas bacitracin (24.8%), chlortetracycline (23.9%), and florfenicol (17.4%) were the most common in pig formulations. The survey-adjusted total content of antimicrobials present in feeds was 25.7 mg/kg [95% CI 19.1–32.4] for chicken and 62.3 mg/kg [95% CI 54.6–70.0] for pig feed formulations.

### Antimicrobials Used to Produce Chicken and Pig Meat

**Figure 1:**
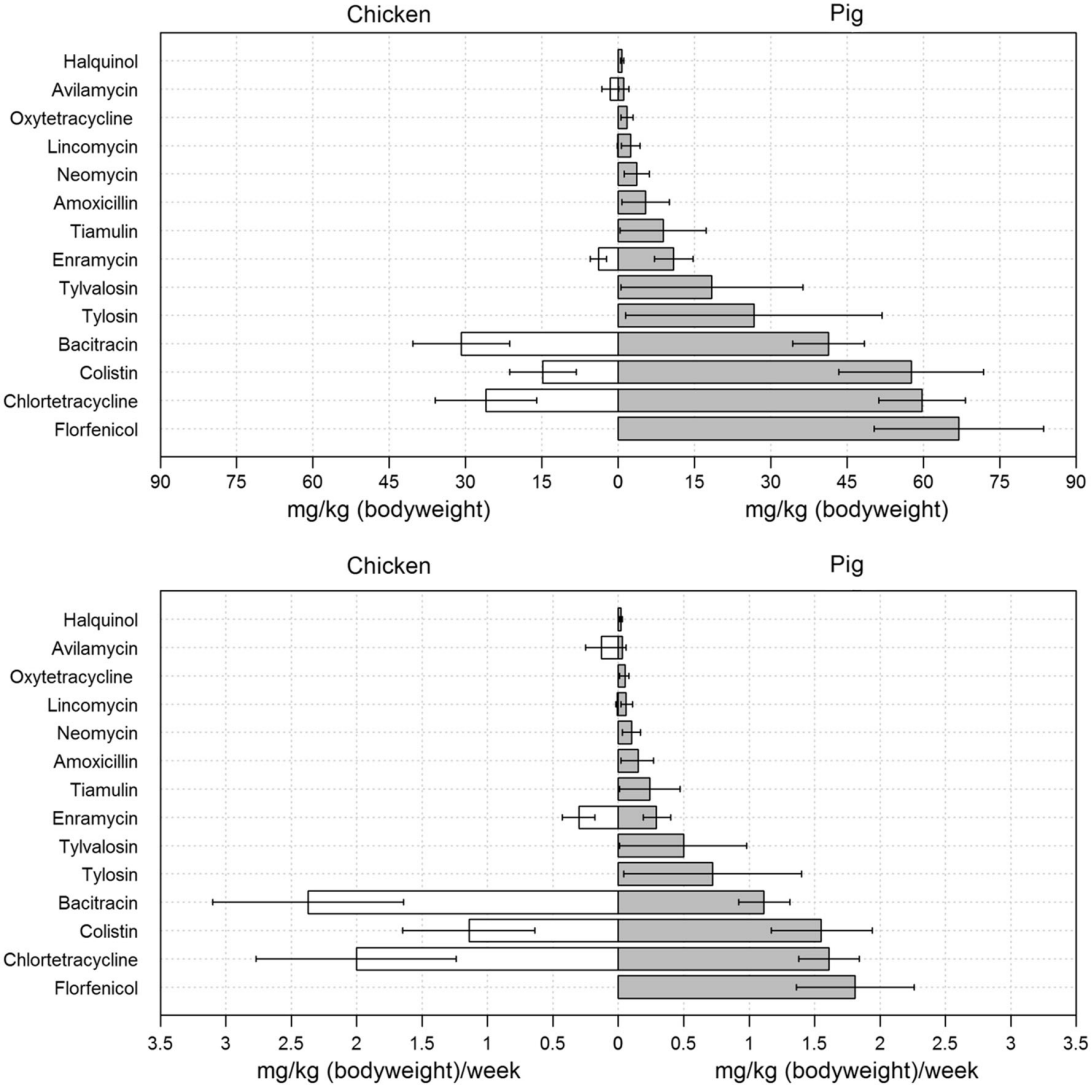
Estimated antimicrobial consumption among Vietnamese chickens and pigs raised with commercial feed rations. The* bars* represent results (average) obtained from 100,000 simulations. * Black lines* represent 95% confidence intervals resulting from the variable probability of inclusion of commercial feeds with each specific antimicrobial

The estimated amounts of in-feed antimicrobials required to raise one chicken and one pig based on production parameters were, respectively, 137.4 mg [95% CI 85.3–189.2] and 26,178 mg [95% CI 17,772–38,692]. The amounts of antimicrobial included in feeds required to raise one kilogram of chicken and one kilogram of pig were 77.4 mg [95% CI 48.1–106.8] and 286.6 mg [95% CI 191.6–418.3], respectively. The calculated amounts of antimicrobial used per kilogram of live animal produced per week were 6.0 mg [95% CI 3.70–8.20] for chickens and 8.2 mg [95% CI 5.2–11.3] for pigs. Bacitracin, chlortetracycline, colistin and enramycin were quantitatively the most commonly used antimicrobials used per kilogram of chicken raised (30.8, 26.0, 14.8, and 3.9 mg, respectively), whereas florfenicol, chlortetracycline, colistin, and bacitracin were quantitatively the most common antimicrobials given to pigs (66.9, 59.7, 57.5, and 41.2 mg, respectively). The estimated antimicrobial consumption to raise pigs and chickens is graphically displayed in Fig. 1.

### Overall Antimicrobial Consumption in Feeds for Chicken and Pig Production

**Figure 2:**
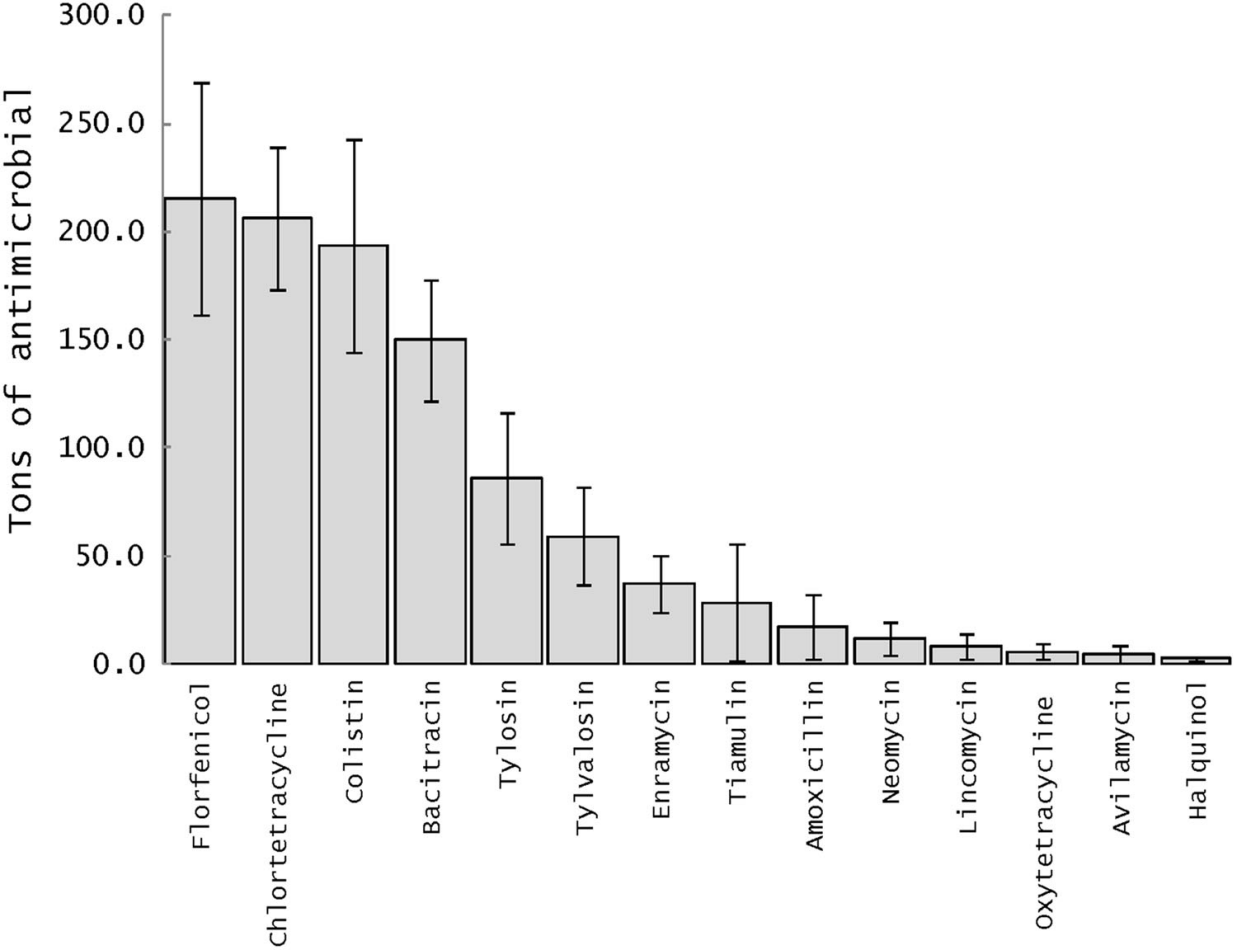
Quantitative estimates of antimicrobials used in chicken and pig production in Vietnam based on animal production figures and survey results

The overall estimated amounts of antimicrobials were 1023.5 tons [95% CI 642.8–1404.2], and 42.2 [95% CI 26.2–58.2] and 981.3 tons [95% CI 616.5–1346.0] for Vietnamese chicken and pig production, respectively. Quantitatively, the most used antimicrobials were, in decreasing order, florfenicol (215.2 tons), chlortetracycline (206.2 tons), colistin (193.3 tons), bacitracin (149.6 tons), and tylosin (85.7 tons). All other antimicrobials were used in quantities less than 40 tons (Fig. 2).

Quantitatively, 57.0% of total usage (583.6 tons) corresponded to antimicrobials regarded by WHO as of ‘critical importance,’ ‘of high importance,’ or ‘of importance’ for human medicine, including amoxicillin, colistin, chlortetracycline, neomycin, oxytetracycline, chlortetracycline, and bacitracin. Such antimicrobials represented 92.6% (39.1 tons) and 55.5% (544.5 tons) of total usage for chicken and pig production, respectively.

## Discussion

Our results indicate greater amounts of in-feed antimicrobials to raise one kg of live pig (286.6 mg) compared with one kg of live chicken (77.4 mg) and, overall, a much higher magnitude of in-feed usage of antimicrobials in pig production compared with chicken production in Vietnam (981.3 tons vs. 42.2 tons, respectively). This is a reflection of (1) a higher antimicrobial content in pig rations (62.3 mg vs. 25.6 mg per kg of feed); (2) the relatively higher feed conversion factor of the pig species (i.e., a higher amount of feed required to produce one kilogram of live animal); and (3) higher levels of pig production, since in Vietnam pork is the main type of meat consumed (35.0 kg per capita per year vs. 12.4 kg of poultry meat) (FAO 2015). There is a scarcity of global data on the magnitude of use of AGP in animal production. A recent study estimated that ~690 and ~350 mg of antimicrobials were, respectively, used to raise one kg of pork and poultry in the USA. However, in that study the fraction of the reported usage corresponding to in-feed antimicrobials was not reported (Krishnasamy et al. 2015).

The overall annual estimates of usage in feeds aimed at pig and chicken production (~1023 tons) represent 1.6% of the global estimate of ~63 thousand tons of antimicrobials used for animal production worldwide (Van Boeckel et al. 2015). Although we need to take these figures with caution, these estimates appear to be high in relation to the Vietnam’s population (90 million, or ~1.2% of the world’s population), and given that our estimates exclude any other additional antimicrobials supplemented by the farmers to prevent or treat disease.

Our results suggest that in Vietnam antimicrobials used in feed represent ~15–20% of total antimicrobial usage in chicken production, based on data from a recent survey in the Mekong Delta, where farmers used 690 mg per chicken raised, mostly by adding them in water (Carrique-Mas et al. 2014). Unfortunately, quantitative data on therapeutic and prophylactic antimicrobial usage in pig production are not currently available. Further research efforts are required to fill this important knowledge gap.

The data used in this study have substantial limitations. For example, the sales of each feed product were unknown and some of the formulations were marketed for specific production types (i.e., starter ratio, pig finishing, layer hens, etc.), although this information was not consistently available. In the absence of this information, we chose to correct for the companies’ geographical presence, assuming that sales of individual products were proportional to the companies’ geographical scope, which may not be accurate. Because of this, results have to be interpreted with caution.

We chose to derive antimicrobials consumed in feed from estimated animal production figures, rather than from feed production figures. An industry report (2010) on animal feeds produced in Vietnam indicate a higher overall amount of feeds used (15760 and 2588 thousand tons for pigs and chickens, respectively) vs. our estimates (12550 and 1553 thousand tons for pigs and chickens, respectively). These differences may lead to an underestimation of antimicrobials in feed in our study.

Initial studies carried out several decades ago clearly pointed out important benefits of the use in AGP in terms of overall health and productivity (Dibner and Richards 2005). However, more recent studies have questioned their efficacy in farms managed with higher levels of hygiene and sanitation (Holman and Chenier 2013; Laxminarayan et al. 2015). Countries across the world have variable policies with regard to the use of AGP, ranging from full ban (i.e., European Union countries, Taiwan), to no specific restrictions for the inclusion of antimicrobials in feed (Maron et al. 2013). In Vietnam, antimicrobials can be included in feed formulations subject to approval from the Ministry of Agriculture and Development for each specific commercial product (MARD 2012).

A quantitatively high percentage of antimicrobials administered to chickens and pigs (92.6% and 55.5%, respectively) are listed as ‘important,’ ‘very important,’ or ‘critically important’ by WHO (2011). The use of high amounts of colistin is of concern, given that this antimicrobial is regarded as a last resort antimicrobial in patients with multi-resistant gram-negative infections (Le Minh et al. 2015). Recent reports indicate the emergence of *mcr*-1 plasmid-mediated colistin resistance in China (Liu et al. 2016). This gene has also been very recently identified in Vietnamese pigs (Malhotra-Kumar et al. 2016). In addition to its inclusion in commercial feed rations, colistin is widely used in Vietnam and other countries by chicken and pig farmers for prophylactic and therapeutic purposes (Dang et al. 2013; Carrique-Mas et al. 2014). It is suspected that this usage may play a role in selection for the transmission of the *mcr*-1 gene (Skov and Monnet 2016).

Although antimicrobial resistance against tetracyclines is extensive in enteric bacteria in Vietnam (Nhung et al. 2014; Tu et al. 2015) and elsewhere in Asia (FAO 2014), these antimicrobials are still widely used in Vietnam and elsewhere. Some researchers claim that they are still effective in controlling chronic respiratory infections (Del Pozo Sacristan et al. 2012). The inclusion of this antimicrobial in poultry feeds is of particular concern because of potential residues in eggs (Kodimalar et al. 2014). However, in our study it was not possible to determine whether this antimicrobial was included in feed rations aimed at meat or egg chicken production.

The use of tylosin in pig feeds has been shown to lead to an increase in potentially zoonotic erythromycin-resistant enterococci and staphylococci (Aarestrup and Carstensen 1998). This antimicrobial has considerable impact on the generation of genes that code for cross resistance against macrolides, lincosamides, and streptogramin B (Holman and Chenier 2013). Bacitracin is an antimicrobial often used in ointments to treat skin infections (Spann et al. 2004). Recently, there have been concerns against resistance against this antimicrobial in enterococcal, streptococcal, and staphylococcal human infections (Malhotra-Kumar et al. 2003; Yoshida et al. 2011; Gebhard et al. 2014). This antimicrobial has recently been included in the list of banned antimicrobials published by the Vietnamese government, to be enforced from July 2015 (MARD 2014).

In summary, we investigated the types and quantities of antimicrobials used in feeds aimed at Vietnamese pig and poultry production, and from these data we estimated overall consumption of antimicrobials through feed. Results clearly indicate that pig production use higher amounts of antimicrobials in feed compared with chicken production. These data aim to stimulate discussion as well as provide a baseline for further studies on antimicrobial use in this rapidly emerging economy. We recommend the progressive introduction of restrictions in the use of certain antimicrobials in feed, especially those that are regarded of high/critical importance by WHO. It would also be desirable to monitor the sales of antimicrobials aimed in animal production in Vietnam by additional requirements to the veterinary drug industry and feed mills. These changes should be introduced alongside guidelines to improve disease control by other means such as better hygiene and biosecurity on farms.
